# Synergistic Effect of Biochar, Phosphate Fertilizer, and Phosphorous Solubilizing Bacteria for Mitigating Cadmium (Cd) Stress and Improving Maize Growth in Cd-Contaminated Soil

**DOI:** 10.3390/plants13233333

**Published:** 2024-11-28

**Authors:** Wenjun Ma, Panjun Luo, Sarfraz Ahmed, Hafiz Saqib Hayat, Shakeel Ahmad Anjum, Lili Nian, Jun Wu, Yuzhen Wei, Wenxue Ba, Fasih Ullah Haider, Liqun Cai

**Affiliations:** 1 College of Resources and Environmental Sciences, Gansu Agricultural University, Lanzhou 730070, China; mawenjun0104@163.com (W.M.); luopj2021@163.com (P.L.); wujun@gsau.edu.cn (J.W.); feinibuke19851026@163.com (Y.W.); 15809746193@163.com (W.B.); haider281@iga.ac.cn (F.U.H.); 2 Key Laboratory of Dry Land Crop Science, Gansu Agricultural University, Lanzhou 730070, China; 3 Key Laboratory of Remote Sensing, Northeast Institute of Geography and Agroecology, Chinese Academy of Sciences, Changchun 130102, China; sarfraz080@gmail.com; 4 Department of Agronomy, Faculty of Agriculture & Environmental Science, Muhammad Nawaz Shareef University of Agriculture, Multan 60000, Pakistan; saqib.hayat@mnsuam.edu.pk; 5 Department of Agronomy, University of Agriculture, Faisalabad 38000, Pakistan; shakeel.anjum@uaf.edu.pk; 6 College of Forestry, Gansu Agricultural University, Lanzhou 730070, China; nll18893814845@163.com

**Keywords:** cadmium toxicity, soil properties, plant biomass, photosynthesis, Cd-remediation

## Abstract

Cadmium (Cd) contamination threatens human health and plant growth due to its accumulation in edible parts. The sole application of phosphorus-solubilizing bacteria (PSB), biochar (BC), and phosphorus (P) effectively mitigates Cd’s adverse effects in contaminated agricultural systems. However, further investigation into their combined impacts on Cd toxicity and maize (*Zea mays*) production is essential. This study evaluates the synergistic effects of PSB (10 g kg^−1^ of *Bacillus megaterium*), BC (5% *w*/*w*), and P (0.8 g kg^−1^) on soil properties and the morphological and physiological traits of maize cultivated in agricultural soil contaminated with Cd (20 mg kg^−1^). The study revealed that Cd toxicity negatively impacts soil properties, reducing shoot and root biomass, lowering chlorophyll content, and heightening oxidative stress levels. Conversely, the combined use of P, PSB, and BC markedly improved soil properties, increasing the organic matter by 175.94%, available K by 87.24%, and available P by 306.93% compared to the control. This combination also improved maize growth metrics, with increases in aboveground dry biomass (92.98%), root dry biomass (110.33%), chlorophyll a (28.20%), chlorophyll b (108.34%), and total chlorophyll (37.17%). Notably, the treatment reduced Cd concentrations in maize leaves by 61.08% while increasing soil Cd levels by 31.12% compared to the control group. Overall, the synergistic effect of P-BC-PSB is an eco-friendly strategy for mitigating Cd toxicity in contaminated soil. However, further studies are required to explore its effects and molecular mechanisms on other crops.

## 1. Introduction

Cadmium (Cd) contamination in agricultural ecosystems arises from anthropogenic sources such as phosphate fertilizers, mining, industrial effluents, and pesticides, posing significant environmental challenges globally [1]. Cd is persistent in the environment and can accumulate in cereal crops, with concentrations often exceeding safe limits in contaminated soils. Recent studies have reported that Cd levels can reach up to 2.0 mg kg^−1^ in agricultural soils, particularly in regions with intensive fertilizer application [2]. The distribution of Cd varies widely, influenced by soil properties such as the organic matter (OM), pH, and oxidation–reduction potential, which affect its bioavailability and toxicity to plants [3]. Through its roots, the plant absorbs Cd, which then moves to every organ in the plant [4]. When a plant is exposed to Cd poisoning, it undergoes several physiological changes. Elevated Cd levels lead to decreased photosynthetic pigments, impaired water absorption, disrupted nutrient movement, altered metabolism, and increased oxidative stress [5]. Research shows that Cd disrupts physiological and biochemical processes, hindering plant growth by affecting the balance of reactive oxygen species (ROS), which can cause lipid peroxidation and damage cellular membranes [6]. This imbalance modifies critical signal transduction pathways related to photosynthesis and antioxidant systems [7]. Additionally, Cd toxicity reduces stomatal conductance and overall plant vitality, impacting the photosynthetic system by lowering chlorophyll *a* and *b* and carotenoids [8,9]. However, sustainable and environmentally friendly mitigation strategies for Cd contamination are increasingly becoming the focus of research and development [10].

Chemical chelates like diethylenetriaminepentaacetic acid (DTPA) can enhance the bioavailability of Cd [11]. Still, they may also increase the risk of Cd leaching and negatively impact soil health and integrity [12]. Bioremediation is often utilized alongside other treatment technologies to address Cd contamination due to its effectiveness, reduced energy requirements, and lower risk of secondary pollution [13]. Numerous studies have investigated the relationship between phosphorus (P) deficiency and Cd contamination in soil, highlighting the challenges of addressing both issues simultaneously [14]. Nonetheless, P deficiency and Cd toxicity frequently coexist in agricultural soils [13]. Phosphate-solubilizing bacteria (PSB) enhance the soil P availability of Cd-contaminated agricultural soils by releasing mineral-dissolving compounds, including organic acid anions, protons, hydroxyl ions, and siderophores, along with secreting extracellular enzymes and degrading substrates [15]. Specifically, PSB can convert insoluble P into a form that plants can readily absorb [16]. According to recent studies, PSB may survive on the surfaces of various soil types and the roots and interior tissues of plants [17]. Several studies have highlighted that PSB enhances soil’s chemical and physical characteristics while encouraging plant development, growth, and yield [18]. Furthermore, recent studies have demonstrated that PSB can withstand Cd contamination, reduce toxicity to vegetables and crops, and significantly enhance crop growth in agricultural rhizospheres contaminated with Cd [12,15]. Thus, it is essential to identify effective management strategies that integrate P and PSB to alleviate Cd toxicity and enhance crop yields in contaminated agricultural soils.

Apart from phosphorus-solubilizing bacteria (PSB), biochar (BC) is a carbon-rich material produced through the pyrolysis of biomass, such as agricultural residues, forestry waste, or animal manure [19]. This thermally treated biomass primarily consists of carbon and is characterized by its high surface area, porosity, and stability, making it an effective soil amendment [20]. Biochar can significantly regulate the soil’s environmental variables and positively influence the physical characteristics of the soil [21]. Its porous structure supports the colonization and activity of soil microorganisms by fostering a favorable environment for microbial growth [22]. Furthermore, BC is frequently applied as a remediation strategy for contaminated soils since it can absorb specific Cd ions. However, it is important to note that secondary contamination is possible since environmental elements like acid rain and wet–dry alternation can impact BC’s adsorption ability, potentially releasing the absorbed contaminants back into the soil [18]. Microorganisms can thrive within BC’s porous structure, protecting them from biotic and abiotic stresses [23]. BC can directly or indirectly affect the composition and structure of microbial communities by serving as a nutrient source [20]. Additionally, BC can alter soil pH and nutrient levels, impacting microbial enzyme activity. Thus, the potential for BC to remove Cd from the environment is significant when combined with microbes that solubilize phosphate [24]. Prior research has indicated that BC combined with P and PSB can effectively remove Cd [11,14]. However, the process by which BC, P, and PSB work together to eliminate Cd and enhance microbial communities in the context of Cd-contaminated agricultural soil has yet to be comprehensively examined.

While the individual application of P [25,26], PSB [27], or BC [19] has proven effective in remediating Cd contamination in agricultural soils, their combined effects have yet to be fully explored. Nevertheless, the information about the potential of the synergistic application of P, PSB, and BC to mitigate the toxic effect of Cd and improve crop production in cereals is a promising avenue that needs to be highlighted. Consequently, the current purpose of the experiment was to evaluate the synergistic impacts of P, PSB, and BC on enhancing maize growth, improving P availability, and mitigating Cd toxicity. This study is significant as it offers valuable insights into potential solutions for Cd contamination, fostering engagement within the scientific community and encouraging further research in this field. It was hypothesized that the synergistic effects of P, PSB, and BC would enhance Cd remediation in plants while simultaneously promoting maize growth. This enhancement is anticipated to occur through the mitigation of oxidative stress and the strengthening of the antioxidant defense systems in plants.

## 2. Results

### 2.1. Effect on Soil Properties

The study findings indicated that the application of BC, P, and PSB has a significant positive impact on the OM content, available K, and available P in soil contaminated with Cd (Table 1). Specifically, the presence of PSB increased the available K and P by 20.73% and 47.89%, respectively, compared to the control group. However, the effect on soil OM was non-significant. Meanwhile, BC application resulted in substantial enhancements in soil properties: the OM increased by 114.95%, available K by 28.31%, and available P by 127.46% relative to the control. The application of P also led to improvements in OM (26.05%), available K (14.33%), and available P (47.84%) compared to the control. Regarding soil pH, the results were less pronounced; the maximum soil pH observed was 8.14 in the control group without any amendments, while a lower pH of 7.97 was recorded in soils treated with BC, P, and PSB. In particular, the synergistic application of P, PSB, and BC resulted in remarkable increases in the OM, available K, and available P, with enhancements of 175.94%, 87.24%, and 306.93%, respectively, compared to the control group.

### 2.2. Effect on the Shoot and Root Dry Biomass of Maize

**Figure 1:**
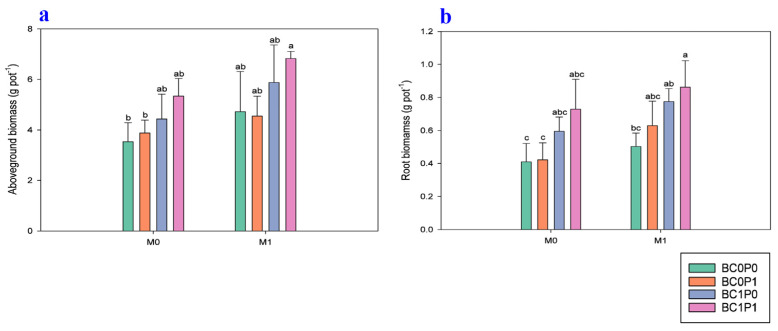
Effect of phosphorous (P), phosphorous-solubilizing bacteria (PSB), and biochar (BC) on the (**a**) root and (**b**) shoot dry biomass of maize grown in cadmium (Cd; 20 mg kg^−1^)-contaminated soil. Note: BC0 (control), BC1 (biochar; 5% *w*/*w* biochar), P0 (0 g kg^−1^ phosphorous), P1 (0.8 g kg^−1^ phosphorous), M0 (0 g kg^−1^ of *Bacillus megaterium*), and M1 (10 g kg^−1^ of *Bacillus megaterium*).

The study findings indicated that the application of BC, P, and PSB has significantly positively improved the aboveground biomass and root dry biomass of maize grown under Cd-contaminated soil (Figure 1). Specifically, the presence of PSB increased the aboveground biomass and root dry biomass of maize by 27.83% and 28.42%, respectively, compared to the control group. Meanwhile, BC application significantly enhanced the aboveground biomass and root dry biomass of maize by 34.61% and 50.64% relative to the control. The application of P also led to an enhancement of the aboveground biomass and root dry biomass of maize by 10.91% and 15.76% compared to the control. Particularly, the synergistic application of P, PSB, and BC resulted in remarkable increases in the aboveground dry biomass and dry root biomass of maize, with enhancements of 92.98% and 110.33%, respectively, compared to the control group.

### 2.3. Effect on MDA, SOD, and CAT Contents in Maize Leaves

**Figure 2:**
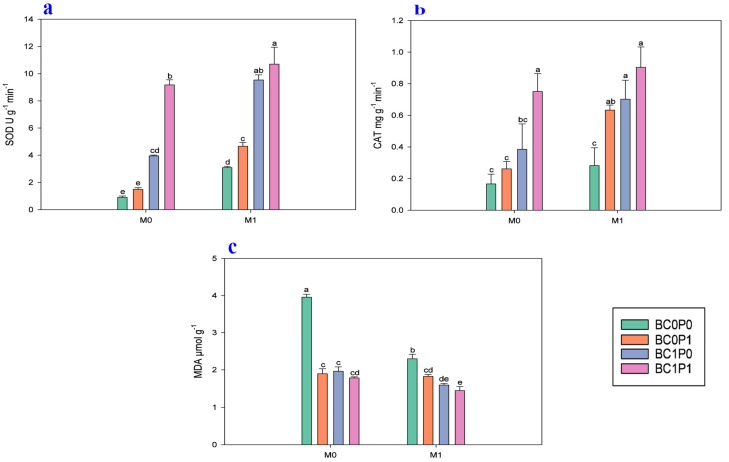
Effect of phosphorous (P), phosphorous-solubilizing bacteria (PSB), and biochar (BC) on the (**a**) malondialdehyde (MDA), (**b**) superoxide dismutase (SOD), and (**c**) catalase (CAT) of maize leaves grown in cadmium (Cd; 20 mg kg^−1^)-contaminated soil. Note: BC0 (control), BC1 (biochar; 5% *w*/*w* biochar), P0 (0 g kg^−1^ phosphorous), P1 (0.8 g kg^−1^ phosphorous), M0 (0 g kg^−1^ of *Bacillus megaterium*), and M1 (10 g kg^−1^ of *Bacillus megaterium*).

The study findings indicated that the application of BC, P, and PSB has a significant impact on the oxidant (MDA) and antioxidant (SOD and POD) activities of maize leaves grown under Cd-contaminated soil (Figure 2). Specifically, the concentration of MDA in maize leaves was higher in controls where maize plants were grown in Cd-contaminated soil. However, the application of BC, P, and PSB significantly reduced the concentration of MDA in maize leaves, with reductions of 46.86%, 41.14%, and 33.88%, respectively, compared to the control group. Particularly, the synergistic application of P, PSB, and BC resulted in a remarkable decrease in the MDA concentration in maize leaves, with a decrease of 173.24% compared to the control group. Similarly, the antioxidant (SOD and POD) activities of maize leaves were significantly improved when plants were grown under Cd-contaminated soil. Specifically, the presence of PSB increased the SOD and POD of maize leaves by 80.31% and 69.57%, respectively, compared to the control group. Meanwhile, BC application significantly enhanced the SOD and POD of maize leaves by 228.61% and 104.16% relative to the control. The application of P also led to an enhancement of the SOD and POD of maize leaves by 48.88 and 57.81% compared to the control. 

### 2.4. Effect on Chlorophyll Content and Photosynthesis Rate of Maize Plants

**Figure 3:**
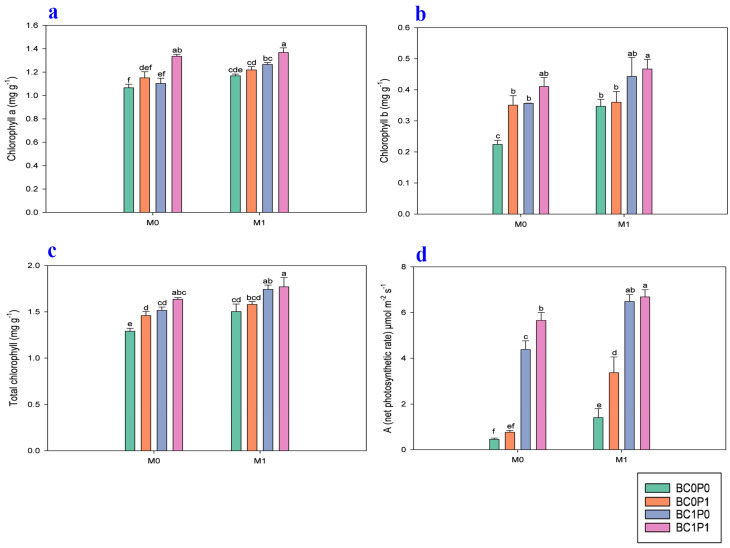
Effect of phosphorous (P), phosphorous-solubilizing bacteria (PSB), and biochar (BC) on the (**a**) chlorophyll *a*, (**b**) chlorophyll *b*, (**c**) total chlorophyll, and (**d**) photosynthesis rate (A) of maize leaves grown in cadmium (Cd; 20 mg kg^−1^)-contaminated soil. Note: BC0 (control), BC1 (biochar; 5% *w*/*w* biochar), P0 (0 g kg^−1^ phosphorous), P1 (0.8 g kg^−1^ phosphorous), M0 (0 g kg^−1^ of *Bacillus megaterium*), and M1 (10 g kg^−1^ of *Bacillus megaterium*).

The study findings indicated that the application of BC, P, and PSB has significantly positively improved the chlorophyll *a*, chlorophyll *b*, total chlorophyll, and photosynthesis rate (A) of maize grown in Cd-contaminated soil (Figure 3). Specifically, the presence of PSB increased the chlorophyll *a*, chlorophyll *b*, total chlorophyll, and photosynthesis rate (A) of maize by 7.82, 20.49, 11.72, and 59.26%, respectively, compared to the control group. Meanwhile, BC application significantly enhanced the chlorophyll *a*, chlorophyll *b*, total chlorophyll, and photosynthesis rate (A) of maize by 10.11, 30.84, 14.28, and 286.99% relative to the control. The application of P also led to an enhancement of the chlorophyll *a*, chlorophyll *b*, total chlorophyll, and photosynthesis rate (A) of maize by 10.19, 15.87, 6.53, and 29.43% compared to the control. Specifically, the synergistic application of P, PSB, and BC resulted in remarkable increases in the chlorophyll *a*, chlorophyll *b*, and total chlorophyll of maize, with enhancements of 28.20%, 108.34, and 37.17%, respectively, compared to the control group.

### 2.5. Effect on Cd Concentration in Maize Leaves, Roots, Shoots, and Soil 

**Figure 4:**
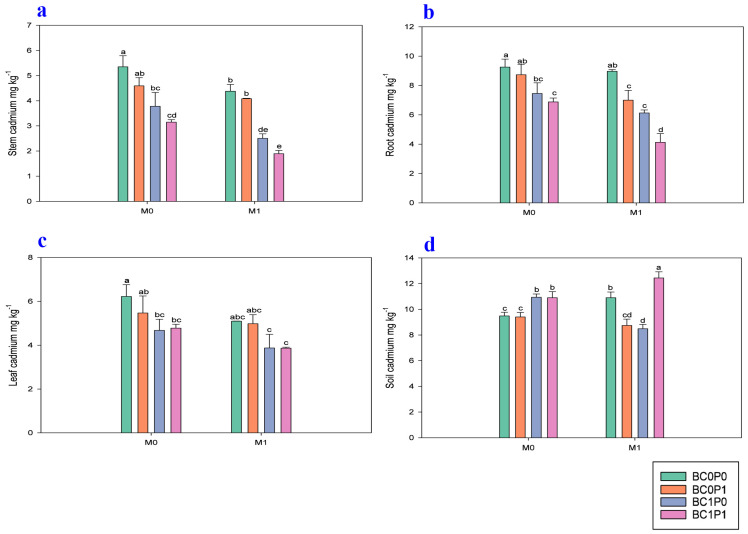
Effect of phosphorous (P), phosphorous-solubilizing bacteria (PSB), and biochar (BC) on the (**a**) shoot, (**b**) roots, (**c**) leaves, and (**d**) soil of maize leaves grown in cadmium (Cd; 20 mg kg^−1^)-contaminated soil. Note: BC0 (control), BC1 (biochar; 5% *w*/*w* biochar), P0 (0 g kg^−1^ phosphorous), P1 (0.8 g kg^−1^ phosphorous), M0 (0 g kg^−1^ of *Bacillus megaterium*), and M1 (10 g kg^−1^ of *Bacillus megaterium*).

The study findings demonstrate that the application of BC, P, and PSB significantly reduced the concentration of Cd in the leaves, shoots, and roots of maize plants grown in Cd-contaminated soil (Figure 4). Specifically, the presence of PSB reduced the Cd levels in maize tissues by 18.72% in leaves, 31.19% in shoots, and 23.23% in roots compared to the control group. Meanwhile, BC was even more effective, achieving reductions of 26.63% in leaves, 62.42% in shoots, and 38.04% in roots relative to the control. The application of P also contributed to minimizing Cd concentrations, with reductions of 4.07% in leaves, 16.68% in shoots, and 18.93% in roots compared to the control. Specifically, the synergistic application of P, PSB, and BC resulted in remarkably decreased concentrations of Cd in the leaves, shoot, and root of maize, with decreases of 61.08%, 182.29, and 124.03%, respectively, compared to the control group. Additionally, the study found that the application of BC and P significantly reduced the bioavailability of Cd in the contaminated soil. Meanwhile, the effect of PSB on the soil Cd levels was non-significant. Specifically, BC minimized the soil Cd bioavailability by 10.97%, whereas P resulted in a smaller decrease of 4.21% compared to the control group. Furthermore, the synergistic application of P, PSB, and BC led to a notable reduction in the soil Cd bioavailability, with an enhancement of 31.12% compared to the control group.

### 2.6. PCA and Correlation Analysis 

**Figure 5:**
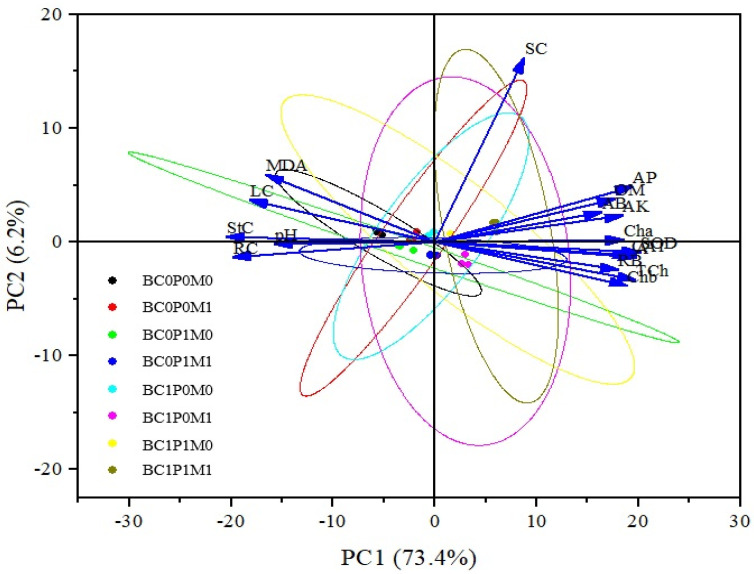
PCA-Biplot of physio-morphological traits of maize. Note: BC0 = control; BC1 = biochar; 5% *w*/*w* biochar; P0 = 0 g kg^−1^ phosphorous; P1 = 0.8 g kg^−1^ phosphorous; M0 = 0 g kg^−1^ of *Bacillus megaterium*; M1 = 10 g kg^−1^ of *Bacillus megaterium*; OM = organic matter; AK = available potassium; AP = available phosphorus; AB = aboveground biomass; RB = root biomass; Cha = chlorophyll a; Chb = chlorophyll b; TCh = total chlorophyll; A = net photosynthetic rate; LC = leaf cadmium; StC = shoot cadmium; RC = root cadmium; SC = soil cadmium.

Based on the correlations of physiological–morphological traits in maize, PCA was employed to examine the associations between various treatments and these traits, The PCA included soil properties and physio-morphological traits, identifying two principal components (PCs) with eigenvalues greater than 1, while the others were deemed less significant. These two PCs accounted for approximately 79.6% of the treatment differences (Table S1, Figure 5). The first PC explained about 73.4% of the total variability, primarily influenced by SOD, the net photosynthesis rate (A), total chlorophyll, and available P. The second PC contributed around 6.2% of the variation and was mainly associated with soil Cd and MDA. The PCA biplot indicated a significant distinction between BC0P0M0 and BC1P1M1 (Figure 5).

**Figure 6:**
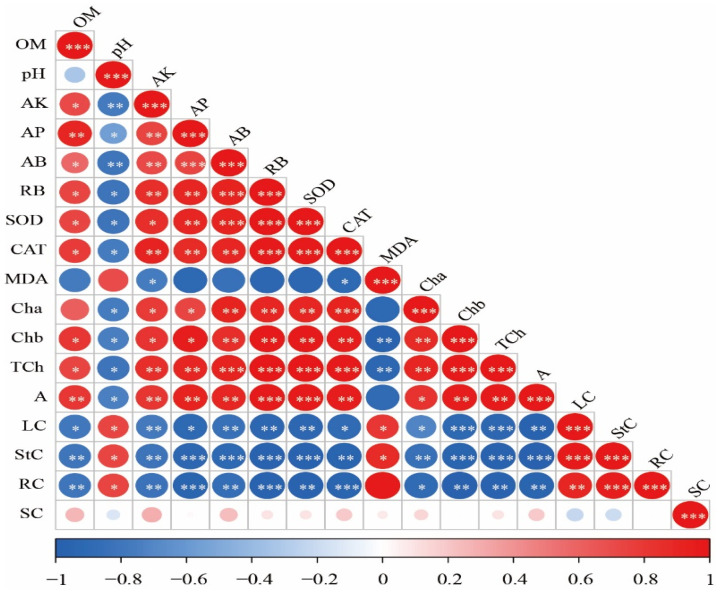
Correlation analysis of physio-morphological traits of maize. Note: *, **, and *** in the figure indicate *p* ≤ 0.05, *p* ≤ 0.01, and *p* ≤ 0.001, respectively. OM = organic matter; AK = available potassium; AP = available phosphorus; AB = aboveground biomass; RB = root biomass; Cha = chlorophyll a; Chb = chlorophyll b; TCh = total chlorophyll; A = net photosynthetic rate; LC = leaf cadmium; StC = shoot cadmium; RC = root cadmium; SC = soil cadmium.

Figure 6 shows a significant positive correlation between available K, available P, aboveground dry biomass, root dry biomass, SOD, CAT, chlorophyll a, chlorophyll b, total chlorophyll, and net photosynthesis rate. Conversely, these indicators correlated significantly negatively with leaf Cd, stem Cd, root Cd, and pH levels. Organic matter also demonstrated a significant positive correlation with available K, available P, aboveground biomass, root biomass, SOD, CAT, chlorophyll b, total chlorophyll, and net photosynthesis rate, while showing a significant negative correlation with leaf Cd, stem Cd, and root Cd. MDA was significantly positively correlated with leaf Cd and stem Cd but showed a significant negative correlation with available K and CAT. Furthermore, there was a significant positive correlation among leaf Cd, stem Cd, root Cd, and soil pH levels. Notably, soil Cd does not exhibit significant correlations with other indicators.

## 3. Discussion

BC, PSB, and P decreased the availability of Cd in contaminated soil and improved maize productivity. In the present study, organic and inorganic amendments in Cd-contaminated soil enhanced the availability and stocks of key soil properties, with the available K increasing by 28.31% and organic matter rising by 114.95%. However, the application of these treatments resulted in a decrease in soil pH to 7.97, compared to a maximum pH of 8.14 in the control group. This reduction in pH is generally considered more favorable for nutrient availability and plant growth under pollution conditions. In alignment with the findings of Beheshti et al. [28] and Wei et al. [29], PSB converts insoluble P in the rhizosphere into soluble forms that plants can utilize, aided by specific metabolites or interactions with other organisms. Moreover, those microbes capable of dissolving phosphates can also immobilize Cd (Figure 4). This can be performed by releasing PO_4_^3−^, which tends to form precipitates of Cd, or by biosorption. Moreover, as a supplement to nutrients, these microbes release compounds that form chelates or change the form of toxic Cd, which reduces the uptake and assimilation of hazardous metals in crops [30,31]. Two phosphate-solubilizing and Cd-resistant bacteria, *Pantoea ananatis* and *Bacillus thuringiensis*, isolated by Liu et al. [20], solubilized insoluble inorganic P and immobilized soil lead (Pb). They improved the availability of P in the soil, while reducing the solubility of Cd in the soil. Moreover, they also enhanced the populations of PSB in the rhizosphere of maize, which coordinately released or bound Cd in the rhizosphere soil and refused to be absorbed by maize.

Soil hardening and water eutrophication are environmental issues linked to the widespread use of conventional P fertilizers [32]. In contrast, PSB fertilizers can gradually release P in the agricultural soil, posing minimal ecological risks [33]. Mendes et al. [34] note that when organic acids are added to the soil, they can reduce soil pH and solubilize inorganic phosphate with aluminum (Al), iron (Fe), magnesium (Mg), and calcium (Ca). Li et al. [35] reported that *Bacillus subtilis* enhances P availability in contaminated soils through the biosynthesis of glycolic acid, which stimulates the activity of acid phosphatase and pyrophosphate. This finding underscores the role of PSB in improving soil nutrient dynamics, particularly in Cd-contaminated environments. The study indicated that the presence of *Bacillus subtilis* can mitigate Cd toxicity by promoting chemical interactions that reduce Cd availability. Specifically, Cd^2^⁺ ions may form precipitates with hydroxide ions (OH^−^) and other mineral phases, thereby decreasing their mobility and bioavailability in the soil. Beesley et al. [36] supported the idea, stating that the pH of the soils was greatly responsible for the kind and quantity of Cd present in the soils. Furthermore, it was discovered that BC’s oxygen-containing functional groups (OFGs) might also be capable of adsorbing Cd [37,38]. Adsorption also occurred via electrical attraction [11], forming a coordination compound with the OFGs. This study also probed the effects of the decrease in stress in Cd-contaminated soils due to BC, P, and PSB on maize chloroplasts, chlorophylls, and root–shoot biomass (Figure 1 and Figure 3). Compared to the control, maize leaves’ *Chl a* and *Chl b* contents increased with each treatment. The increased concentrations of PSB in our study effectively reduced Cd stress and enhanced photosynthetic efficiency in maize plants. As a result, the total chlorophyll content in maize treated with BC and PSB significantly increased. This finding highlights the beneficial role of PSB in improving chlorophyll levels and overall plant health under Cd-contaminated conditions [39].

Interestingly, while the BC, P, and PSB treatments improved maize productivity and reduced Cd stress, an increase in soil Cd concentration was observed in variants treated with all three agents. This phenomenon may be attributed to several factors. First, the application of BC can enhance the solubility of certain metal ions under specific conditions, potentially leading to increased mobility of Cd in the soil [40]. Additionally, the release of organic acids from the microbial activity associated with PSB could alter soil chemistry, facilitating the dissolution of previously immobilized Cd [41]. Furthermore, while these amendments aim to reduce Cd uptake by plants through precipitation and adsorption mechanisms, they may inadvertently increase the concentration of Cd in the soil solution available for plant uptake [42]. This suggests that while BC, P, and PSB can mitigate Cd toxicity in plants, careful consideration is needed regarding their impact on soil Cd levels.

Research indicates that *Bacillus megaterium* can alter the soil microbial community composition and increase the bioavailability of P. For instance, Zhao et al. [43] demonstrated that this bacterium positively affects soil nutrient dynamics, improving cucumber growth by enhancing the availability of essential nutrients like P and K. Similarly, Nascimento et al. [44] highlighted the plant-growth-promoting activities of *Bacillus megaterium*, emphasizing its role in nutrient solubilization and stress resistance. The mechanism by which *Bacillus megaterium* facilitates P release involves the secretion of organic acids and enzymes that dissolve insoluble phosphate compounds, making them more accessible to plants [45]. This process not only benefits plant growth but also contributes to the overall health of the soil ecosystem by promoting beneficial microbial interactions [41]. Furthermore, Jahil and Kamal [46] found that inoculating sunflower (*Helianthus annuus*) plants with *Bacillus megaterium*, along with other organic amendments, led to improved growth and yield, underscoring its effectiveness in nutrient mobilization. In our study, we hypothesize that *Bacillus megaterium* played a crucial role in accelerating the dissolution of P from both the powdered fertilizer and BC, thereby enhancing P availability for maize plants. This synergistic effect is expected to improve maize growth and mitigate Cd stress through enhanced nutrient uptake and reduced oxidative damage.

Compared to the control group, all treatments resulted in a significant increase in the dry weights of both the shoot and root sections of maize, with increases exceeding 100% (Figure 1). More relevant, while plants remediated with BC or PSB separately exhibited significantly inferior and statistically similar dry weights for mature plants to the control, plants with PSB-loaded BC exhibited considerably similar or better dry weights than the control plants. In effect, it was observed that while biochar and PSB are two substances that can enhance maize growth as single agents, their combination leads to a superior response. Studies have established that the IAA produced and released by PGPB can increase the root length and branching at the perimeter zone. As a result, plants could take in more nutrients required for their growth [47]. Since PGPB exhibited enhanced P solubilization capabilities, the readily available P nutrient for plant growth was in plentiful supply. According to the consequence of BC, they have many pores that could enhance soil’s physical characteristics, such as pore size distribution and bulk density. Additionally, plants would receive higher oxygen and water contents, reduced toxicity from Cd, decreased competition from native and useful bacteria, a high PSB survival ratio, and bioactivity [48].

Finally, PSB and biochar are capable of increasing crop yields by improving the microbial community of phosphorus in the rhizosphere and the number of bacteria that are good for plant growth and other potential factors such as P solubilization [49]. Thus, the plants can produce antioxidant enzymes like SOD, CAT, and MDA to lessen the effects of heavy metal stress [50]. These enzymes reduce oxidizing chain reactions [51], a type of oxidative stress [52,53]. When BC, P, and PSB were applied in our experiment, it indicated that these antioxidant enzymes in maize plants were at higher levels than in the control group (Figure 2). This result was consistent with research showing that adding BC to soils containing Ni or Cd increases APX, SOD, and CAT activities in mung beans (*Vigna radiata*) and sunflowers (*Helianthus annuus*), respectively, compared to the control [51]. Ibrahim et al. [54] and Alotibi et al. [55] have identified that due to the promotion of plant health by BC and the capacity to reduce the production of ROS, along with enhancing resistance to heavy metal stress, plants’ antioxidant defense systems have been enhanced. Because organic amendments can change the cell osmotic pressure and reduce the build-up of dangerous free radicals when combined with antioxidants [56], they can enhance the functioning of essential nutrients [57]. 

Furthermore, PCA data indicated that the characteristics of the carrier types BC, P, and PSB, the changes produced in maize physiology, and the changes in the function of the soil were the significant causes of the growth of maize (Figure 5). The choice of the type of biochar dominated the values of PC1, which could explain the portion of the total variability (% by PCA). The BC, P, and PSB were more prominent in PC2, conveying a percentage of total variance. Hence, there was a substantial negative relationship between soil contents, maize MDA, Cd concentrations, and the biomass of the maize (both shoot and root parts). The antioxidant units SOD, CAT, and POD, as well as *Chl a* and *Chl b*, were positively signed. While this study shows promise, its controlled greenhouse conditions may not adequately capture the complexities of field environments, including soil-type and climate variations. The long-term impacts on crop productivity and soil health still need to be assessed, and our findings primarily pertain to maize, necessitating further exploration of other crops. Another limitation of this study was the lack of measurement of the initial total P concentration in the soil. This omission may have prevented a comprehensive understanding of how the initial soil P levels could interact with the added Cd and *Bacillus megaterium*, which is known for its ability to solubilize P compounds. Future studies should prioritize measuring the initial soil total P concentration to better assess its role in mitigating Cd stress on plants and to elucidate potential interactions among these factors. Additionally, investigating the molecular mechanisms of their interactions and exploring other bioremediation strategies could enhance Cd mitigation. Finally, assessing the economic viability of these approaches in commercial agriculture is essential for promoting sustainable practices in Cd-affected regions. Therefore, it is recommended that field studies be conducted in the future to assess the efficacy of BC, P, and PSB combinations.

## 4. Materials and Methods

### 4.1. Experiment Material Collection

The study employed various materials to investigate the impacts of *Bacillus megaterium*, P, and BC on Cd-contaminated soil and maize plants. Soil samples were obtained from Anding District in Dingxi city, Gansu Province, China. After being air-dried and cleaned of stones, leaf residues, root fragments, and other debris, the samples were sieved through a 4 mm mesh and stored for subsequent experiments and soil physical and chemical analyses. The specific characteristics of the soil are detailed in Table 2. The bacteria (*Bacillus megaterium*) used in this study were purchased from Guangxi Nongbao Bioengineering Co., Ltd. The effective viable bacteria count was ≥10 billion/g, and the dosage form was powder, with the characteristics of fast reproduction, strong vitality, safety, and non-toxicity. The test P fertilizer used was superphosphate (P_2_O_5_ ≥ 16.0%) in granular form, purchased from Wengfu (Group) Co., Ltd. (Guiyang, China). The BC utilized in this research was obtained from Liaoning Jinhefu Agricultural Science and Technology Co., Ltd. (Shenyang, China), and its specific properties are shown in Table 2. The experimental maize variety was Longdan No. 4, a late-maturing hybrid bred by the Gansu Academy of Agricultural Sciences with a growth period of 137 days. The seeds were purchased from Gansu Lanzhou Jinxin Agricultural Technology Co., Ltd. (Lanzhou, China).

### 4.2. Experimental Setup

The pot experiment employed a three-factor, three-level orthogonal test design to systematically assess the impacts of BC, P fertilizer, and PSB on maize growth in Cd-contaminated soil. The experimental factors were defined as follows: BC was applied at two levels, 0% and 5% (designated as BC0 and BC1); P fertilizer was tested at levels of 0 g kg^−1^ and 0.8 g kg^−1^ (designated as P0 and P1), corresponding to 0 kg ha^−1^ and 800 kg ha^−1^; and PSB (*Bacillus megaterium*) were included at levels of 0 g kg ^−1^ and 10 g kg^−1^ (designated as M0 and M1), which translates to 0 kg ha^−1^ and 1000 kg ha^−1^. The quantities of BC, P fertilizer, and PSB were calculated based on the amount of potted soil used in the experiment. Eight unique treatment combinations were replicated four times, resulting in a total of 32 pots. The experiment commenced in the second week of May 2021, with soil collection from the Anding District in Dingxi City. Exogenous Cd (20 mg kg^−1^) was artificially introduced into the soil to create contamination conditions. Base fertilizers, BC, P fertilizer, and PSB were incorporated into the soil by mid-May 2021. Following these amendments, maize was planted, and the experiment concluded in the last week of July 2021. The research was conducted in a controlled environment at the School of Resources and Environment, Gansu Agricultural University (GAU) in Lanzhou, China. This facility was equipped with rain shelters and sunshade nets to prevent precipitation from affecting the nutrient availability and to effectively regulate indoor temperatures. The cultivation conditions were maintained at a temperature of 25 °C and a photoperiod of 12 h light/12 h dark, ensuring optimal growth conditions for maize. 

Each pot had a height of 24 cm and a diameter of 20 cm and was filled with 5 kg of dry soil. Cd was introduced into the soil at a 20 mg kg^−1^ concentration using an analytical-grade aqueous solution of CdSO_4_8H_2_O. The soil was turned once during the spiking process to maintain a field moisture capacity of 60–70% over two months. After aging, the soil was thoroughly mixed with base fertilizers applied at rates of nitrogen (N) of 0.03 g kg⁻^1^ and potassium oxide (K_2_O) of 0.13 g kg^−1^; urea served as the N source, while potassium chloride was utilized for K. After the incorporation of base fertilizers, BC (at levels of 0% or 5%), P fertilizer (at levels of either 0 g kg^−1^ or 0.8 g kg^−1^), and PSB (at levels of either 0 g kg^−1^ or 10 g kg^−1^) were added to each pot. Notably, distilled water was added to the PSB one day before their application to ensure thorough mixing before introducing them into the soil with the BC and P fertilizer. After these preparations, the soil mixture was adequately watered to facilitate subsequent maize planting. For planting purposes, high-quality corn seeds were selected; each pot initially received a sowing of ten seeds, which was later thinned to five seedlings per pot after emergence. Following this thinning process, the corn plants were allowed to grow for 80 days before harvesting and sampling commenced. Throughout the experiment, meticulous attention was paid to maintaining the soil moisture levels at 60–70% of field capacity, which was assessed prior to the experiment using soil moisture measurements. Additionally, the temperature conditions within the net room were closely monitored to mitigate any adverse effects on maize growth due to excessive heat.

### 4.3. Observations and Methods 

#### 4.3.1. Analysis of Soil Physical and Chemical Properties

Soil organic matter (OM) was quantified using the potassium dichromate-concentrated sulfuric acid external heating method, which effectively measures the OM content [58]. To assess soil pH, samples were mixed with distilled water at a 1:2.5 ratio and measured with a pH-10 acidometer for accurate acidity or alkalinity readings. The available P was extracted using a 0.5 mol L^−1^ sodium bicarbonate solution and quantified through the molybdenum-antimony colorimetric method. The available K was determined by placing 0.5 g of dried soil in a 100 mL triangular bottle, followed by the addition of 50 mL of 1 mol L^−1^ neutral ammonium acetate (NH_4_OAc) solution [59]. The mixture was then oscillated for 30 min to facilitate extraction. After filtering through qualitative filter paper, the filtrate was analyzed using a flame photometer alongside standard potassium solutions for accurate quantification. 

#### 4.3.2. Determination of Dry Plant Aboveground Biomass and Dry Belowground Biomass

In each basin, during ripening, three typical strains of consistent longevity were selected from the stem base according to different test treatments to preserve the integrity of the stem, leaves, and shafts. The cut samples were wrapped in plastic and labeled before being brought indoors for weighing. The underground portion of the corn was thoroughly washed, and the root system was collected, weighed, and recorded. To determine the dry biomass, samples were dyed for 30 min in an oven at 105 °C, followed by drying at 65 °C for an additional 30 min. The moisture content was then assessed, and the dry biomasses (g pot^−1^) of both the aboveground and belowground corn parts were calculated.

#### 4.3.3. Determination of Oxidative and Antioxidative Stress in Maize Leaves

To assess the physiological indicators of corn, leaves exhibiting uniform color and growth were selected. These leaves were washed 2–3 times with distilled water, and excess moisture was absorbed using filter paper. Measurements of various physiological indicators were conducted immediately after washing. For certain analyses, liquid nitrogen was used to freeze the leaves to facilitate rapid subsequent measurements. A 0.5 g plant tissue sample was weighed and ground thoroughly in liquid nitrogen for enzyme activity assays. Subsequently, 5 mL of 0.1 mol mL^−1^ phosphate buffer (pH 5.5–8.8) containing 1% polyvinylpyrrolidone (PVP) was added to the ground tissue and transferred to a 5 mL centrifuge tube. The mixture was centrifuged at 10,000 rpm for 20 min at 4 °C to obtain the crude enzyme extract. Superoxide dismutase (SOD) activity was measured using the nitroblue tetrazolium (NBT) reduction method, where one unit (U) of enzyme activity is defined as the inhibition of photochemical reduction by 50% per unit time [60]. The enzyme activity was calculated based on the protein content of the sample. Similarly, catalase (CAT) activity was determined using the ultraviolet absorption method, where one unit (U) of enzyme activity is defined as the amount of enzyme that reduces the absorbance at 240 nm by 0.01 per minute [61]. Similar to SOD and CAT, activity was also calculated based on the protein content of the sample. The malondialdehyde (MDA) content was assessed using the thiobarbituric acid (TBA) method [62]. In this procedure, a 0.5 g fresh plant sample was weighed and placed in a mortar with 5 mL of a 100 g L^−1^ trichloroacetic acid (TCA) solution. The mixture was ground and homogenized, followed by centrifugation for 20 min at 4 °C at 10,000 rpm to collect the supernatant for further analysis. A volume of 2.0 mL of supernatant was mixed with 2 mL of TCA solution as a control and then combined with 2 mL of a 0.67% TBA solution, followed by boiling in a water bath for 20 min. After cooling, the mixture was centrifuged again, and absorbance was measured at wavelengths of 450 nm, 532 nm, and 600 nm, with three replicates performed alongside blank measurements.

#### 4.3.4. Chlorophyll Content and Photosynthesis Rate

The chlorophyll content was determined using acetone extraction and spectrophotometry [63]. A sample of 0.5 g of fresh corn leaves was ground with a small amount of calcium carbonate and quartz sand in the presence of 3 mL of pure acetone to create a homogenate. Following this, 5 mL of 80% acetone was added and the mixture was transferred to a centrifuge tube for centrifugation at 4000 rpm for 10 min. The supernatant was collected and diluted to a final volume of 10 mL with 80% acetone. A portion of this pigment extract was then diluted further with an additional 4 mL of 80% acetone and transferred to a colorimetric cup for absorbance measurement at wavelengths of 663 nm and 645 nm, using acetone as a blank control. The photosynthetic rate of maize was measured between 9:00 and 11:00 a.m. Pots with maize exhibiting similar growth conditions were selected for each treatment, ensuring that five leaves with comparable potential and color were used to determine the net photosynthetic rate using a GFS-3000 photosynthesis-fluorescence measurement system (WALZ, Effeltrich, Germany) [11]. The collected data were stored for further analysis.

#### 4.3.5. Determination of Cd Contents

To determine the Cd content in maize, plants were harvested and divided into three components: roots, stems, and leaves. Each component was dried in an oven at 105 °C for 30 min, followed by further drying at 80 °C until a constant weight was achieved. The dried samples were then ground and prepared for analysis. Microwave digestion was performed using approximately 0.5 g of the dried plant samples in microwave digestion tubes, treated with 8 mL of concentrated nitric acid (65–68%) and 1 mL of hydrogen peroxide (30%). The samples were digested at escalating temperatures, starting at 150 °C for 10 min, increasing to 190 °C for 25 min, and finally reaching 200 °C for an additional 20 min. After digestion, residual acid was evaporated on a hot plate at approximately 170 °C until the solution volume was reduced to about 1 mL. The resulting solution was diluted to a final volume and stored for subsequent Cd analysis [64]. 

### 4.4. Statistical Analysis

Data observed in this study were analyzed using SPSS 19.0 (IBM, Armonk, NY, USA). To evaluate the impact of Cd on maize’s physiological and growth responses, an analysis of variance (ANOVA) was performed across various treatments. Tukey’s test was employed for multiple comparisons to assess significance (*p* < 0.05), with results presented as means ± standard deviation (SD) from four replicates, where different letters indicate significant differences at *p* < 0.05. Correlation analysis was conducted using R 4.1.1, and principal component analysis (PCA) examined relationships between growth parameters and physiological traits, with significance thresholds set at *p* < 0.05 and *p* < 0.01.

## 5. Conclusions

This study demonstrates that the synergistic application of BC, P, and PSB effectively reduces Cd toxicity and promotes maize growth in contaminated soils. The findings reveal significant improvements in soil properties, including increased OM, available K, available P, and enhanced chlorophyll content and biomass in maize. Furthermore, applying these amendments reduced Cd concentrations in maize tissues while increasing soil Cd levels, indicating a potential strategy for managing Cd contamination. Despite these encouraging findings, additional research is needed to assess the lasting effects on soil quality and agricultural yield across various farming environments. Future research should investigate the molecular mechanisms driving these interactions and evaluate the cost-effectiveness of such strategies in commercial agriculture to enhance sustainable practices in regions affected by Cd.

## Figures and Tables

**Table 1:** Effect of phosphorous (P), phosphorous-solubilizing bacteria (PSB), and biochar (BC) on soil pH, organic matter, available phosphorous, and available potassium of soil contaminated with cadmium (Cd; 20 mg kg^−1^).

Biochar	Phosphorous	Phosphorus-Solubilizing Bacteria	Soil pH	Organic Matter (g kg^−1^)	Available Phosphorous (mg kg^−1^)	Available Potassium (mg kg^−1^)
BC0	P0	M0	8.14 ± 0.01 a	15.35 ± 0.60 de	27.37 ± 0.48 d	318.67 ± 19.40 c
BC0	P1	M0	8.13 ± 0.02 ab	21.69 ± 0.89 c	31.28 ± 0.33 d	345.33 ± 85.78 bc
BC0	P0	M1	8.04 ± 0.06 bcd	11.98 ± 0.14 e	26.72 ± 0.54 d	414.33 ± 13.05 bc
BC0	P1	M1	8.01 ± 0.03 cd	18.12 ± 1.74 cd	40.04 ± 0.91 c	474.67 ± 34.53 ab
BC1	P0	M0	8.07 ± 0.03 abc	33.89 ± 0.31 b	44.75 ± 0.19 c	467.67 ± 31.72 ab
BC1	P1	M0	8.07 ± 0.02 abc	35.74 ± 0.68 b	62.27 ± 0.79 b	474.67 ± 34.53 ab
BC1	P0	M1	8.03 ± 0.04 cd	32.33 ± 1.71 b	66.85 ± 2.27 b	453.67 ± 65.31 b
BC1	P1	M1	7.97 ± 0.02 d	42.37 ± 3.24 a	111.40 ± 4.75 a	596.67 ± 15.53 a

Note: BC0 (control), BC1 (biochar; 5% *w*/*w* biochar), P0 (0 g kg^−1^ phosphorous), P1 (0.8 g kg^−1^ phosphorous), M0 (0 g kg^−1^ of *Bacillus megaterium*), and M1 (10 g kg^−1^ of *Bacillus megaterium*). Each value represents the average of three replicates ± SD (standard deviation), and different letters (a, b, c, d) are significantly different at *p* ≤ 0.05 using the Least Significant Difference Test (LSD). The same acronyms apply in subsequent figures.

**Table 2:** Physical and chemical properties of soil and biochar used in current study.

Properties	Soil	Biochar
Pyrolysis temperature	-	500 °C
Organic matter	13.67 g kg^−1^	-
Carbon	-	53.28%
Total nitrogen	1.12 g kg^−1^	1.04%
Total potassium	-	0.26%
Total phosphorus	-	0.51%
pH	7.97	9.21
Electrical conductivity	3.65 (ms cm^−1^)	-
Calcium	-	0.80%
Magnesium	-	0.47%
Available phosphorus	28.36 mg kg^−1^	-
Available potassium	295.25 mg kg^−1^	-
Ash content	-	35.64
Specific surface area	-	11.3 m^2^ g^−1^
Cd concentration	0.3119 mg kg^−1^	-

## Data Availability

The original contributions presented in the study are included in the article/supplementary material, further inquiries can be directed to the corresponding author.

## Supplementary Materials

The following supporting information can be downloaded at: https://www.mdpi.com/article/10.3390/plants13233333/s1.
