# Supplementary material for: Synergistic Effect of Biochar, Phosphate Fertilizer, and Phosphorous Solubilizing Bacteria for Mitigating Cadmium (Cd) Stress and Improving Maize Growth in Cd-Contaminated Soil
Source: Plants (Basel). 2024 Nov 28;13(23):3333. doi: 10.3390/plants13233333 (PMC11644098; doi:10.3390/plants13233333)
Supplement: Supplementary file 1 [file plants-13-03333-s001.zip › plants-3296005-supplementary.pdf]

Table S1. Extracted Eigenvalues and latent vectors of physio-morphological traits of maize associated with the first three principal components.

Variable	Principal Components	
	**PC1**	**PC2**
Extracted Eigenvalue	12.48	1.06
Percentage of Variance (%)	73.41	6.22
Cumulative (%)	73.41	79.62
**Physio-morphological traits**	**Latent vectors**	
AP	0.25941	0.24538
SOD	0.26957	-0.04626
CAT	0.25675	-0.05809
Chb	0.25167	-0.19243
TCh	0.2628	-0.164
A	0.26375	-0.0688
SC	0.11771	0.81109
MDA	-0.22175	0.29547
